# Integrative Analysis of Antennal Morphology and Olfactory Receptor Gene Expression Across the Three Castes of *Bombus terrestris* (Hymenoptera: Apidae)

**DOI:** 10.3390/insects17010055

**Published:** 2026-01-01

**Authors:** Yu Zhang, Lina Guo, Yuan Guo

**Affiliations:** 1 College of Animal Science, Shanxi Agricultural University, Jinzhong 030801, China; 15183537865@163.com; 2 Shanxi Key Laboratory of Animal Genetic Resource Discovery & Precision Breeding, Jinzhong 030801, China; 3 College of Horticulture, Shanxi Agricultural University, Jinzhong 030801, China

**Keywords:** *Bombus terrestris*, olfactory system, antennal sensilla, olfactory receptor genes, social division of labor, adaptive evolution

## Abstract

To systematically investigate how the olfactory system of *Bombus terrestris* adapts to its social division of labor and reproductive strategies, this study integrated the micromorphology of antennal sensilla and the expression profiles of olfactory receptor (OR) genes from the heads of its three castes (workers, drones, and queens) for a multi-level analysis. Scanning electron microscopy (SEM) revealed that drones possess significantly longer chaetic sensilla (Sch), sensilla trichodea (Str A/B), and sensilla basiconica (Sba A), as well as larger-diameter sensilla coeloconica (Sco) compared to workers and queens, indicating structural and functional specialization for sensitive detection of single key signals (e.g., queen pheromones). In contrast, workers and queens exhibited a more complete composition of sensilla types and a higher sensilla distribution density, suggesting the construction of a perceptual system capable of processing multiple chemical signals simultaneously. RNA-seq combined with qRT-PCR confirmed the significant upregulation of seven OR genes (e.g., *BterOR3*, *BterOR4*) in drones, while workers showed upregulation of *BterOR3*/*5*/*7* accompanied by enrichment of P450 detoxification pathways. Phylogenetic analysis suggested that *BterOR5* serves as a conserved co-receptor, and some OR genes may originate from recent duplication events. In summary, distinct differences were observed in the morphological structure and molecular expression of the olfactory system among *B. terrestris* castes. Drones exhibited structural and gene expression features consistent with specialization in queen pheromone detection, while workers and queens demonstrated sensilla diversity and olfactory receptor expression patterns indicative of a broader response capacity to diverse chemical signals. These findings support the view that the olfactory system has undergone multi-level adaptive evolution driven by social division of labor and reproductive roles.

## Simple Summary

This study investigates the adaptive differentiation of the olfactory system across *Bombus terrestris* castes through integrated morphological and transcriptomic analyses. Drones exhibit a “specialized” strategy characterized by elongated sensilla and upregulation of specific receptor genes for accurate queen pheromone detection, whereas workers and queens employ a “generalist” strategy featuring high sensilla density and balanced gene expression profiles to enable parallel processing of diverse chemical signals. These findings provide novel insights into the evolutionary mechanisms underlying chemical perception in social insects.

## 1. Introduction

In social insects, individuals cooperate through division of labor to perform collective tasks such as brood care and defense, with chemosensation playing a central role in regulating recognition and communication among colony members [1]. Chemical signals are primarily detected by insects through two main chemosensory mechanisms: olfaction, which is responsible for detecting volatile molecules and mediates long-distance communication, and gustation, which recognizes soluble substances and regulates behaviors such as feeding and oviposition [2]. As one of the most important sensory organs in insects, the antennae carry multiple functions including olfaction, touch, and temperature–humidity perception. Their input signals constitute one of the most fundamental sensory pathways in the central nervous system [3,4]. Olfactory receptor neurons (ORNs) can specifically recognize chemical cues related to food, oviposition sites, and mates, thereby activating downstream neural circuits and triggering corresponding behaviors [5].

Hymenoptera (comprising sawflies, wasps, ants, and bees) represents one of the four major hyperdiverse insect groups. As parasitoids, predators, and pollinators, they play critical roles in terrestrial ecosystems and possess significant economic value [6]. The antennal sensilla structures in this order often exhibit remarkable sexual dimorphism corresponding to their ecological roles. For instance, in Pteromalidae [7], differences in sensilla between sexes may be associated with distinct host-location strategies; in Torymidae [8], the sex-specific differentiation of sensilla provides crucial morphological clues for understanding their host-searching behavior; whereas in Bethylidae [9], the sensilla on female antennae may be specialized for short-range host detection, while male antennae appear adapted for long-range mate location. Correspondingly, their olfactory systems also demonstrate significant differentiation at the molecular level. A notable example is observed in ants, where distinct caste-specific differences exist in odorant receptor gene expression; in *Camponotus floridanus* (Buckley, 1866) (Hymenoptera: Formicidae) and *Harpegnathos saltator* (Jerdon, 1851) (Hymenoptera: Formicidae), nearly all olfactory receptors (ORs) are expressed in worker ants, while males express only approximately one-third of these receptors [10]. Bumblebees (Hymenoptera: Apidae), being truly eusocial and generalist-feeding pollinators of considerable ecological importance, utilize chemosensory mechanisms to mediate both plant–pollinator interactions and intranidal social communication [11]. *Bombus terrestris* (Linnaeus, 1758) (Hymenoptera: Apidae), serving as a vital ecological pollinator for numerous crops, has attracted substantial research attention regarding the behavioral and physiological aspects of olfactory systems across different castes (queens, workers, and males) [12]. However, a systematic comparative analysis of caste-specific differentiation in both antennal sensilla ultrastructure and OR gene expression profiles remains lacking. This knowledge gap hinders comprehensive understanding of the structural and functional integration underlying chemical communication mechanisms in their social behaviors.

To systematically investigate the ultrastructural characteristics of antennal sensilla in different castes of *B. terrestris* within the context of sex and division of labor variations, this study first employed scanning electron microscopy (SEM) for high-resolution morphological examination of worker, male, and queen antennae. Subsequently, we compared the expression profiles of OR genes in the head tissues (including antennae) across these three castes through high-throughput transcriptome sequencing. By integrating morphological and molecular biological data, this research aims to elucidate the structural and functional adaptations of the olfactory system in *B. terrestris* under different sexes and social roles, thereby providing new experimental evidence for deeper understanding of caste-specific regulatory mechanisms in olfactory pathways.

## 2. Materials and Methods

### 2.1. Test Insects

The *B. terrestris* (workers, males, and queens) used in this study were provided by the Experimental Apiary of the College of Animal Science, Shanxi Agricultural University.

### 2.2. Sample Preparation and Observation Methods

Ten healthy and intact workers, males, and queens were randomly collected from healthy and robust colonies of *B. terrestris*. Antennae from both sides of each individual were included as experimental samples. Under a stereomicroscope, antennae were completely detached at the scape and subsequently fixed with 2.5% glutaraldehyde solution for 24 h. After fixation, the samples were washed three times with PBS phosphate buffer, each wash lasting 10 min. This was followed by sequential dehydration through an ethanol gradient series of 30%, 50%, 70%, 80%, 90%, 95%, and 100%, with each step maintained for 15 min. The 100% ethanol dehydration step was repeated twice. The dehydrated samples were subjected to critical point drying using a JED-320 freeze dryer (JEOL Ltd., Tokyo, Japan). Each sample was mounted on an aluminum stub with conductive carbon adhesive tape and sputter-coated with a 10-nm gold layer using an SBC-12 coating system (KYKY Technology Co., Ltd., Beijing, China) to ensure optimal surface conductivity. The metallized samples were examined under high vacuum conditions with a JSM-7800F field emission scanning electron microscope (JEOL Ltd., Tokyo, Japan), and images were acquired at an accelerating voltage of 5–20 kV.

Additionally, six workers, males, and queens were respectively selected, and their antennae were excised. The antennae were rinsed three times with 75% ethanol, air-dried at room temperature (25 ± 1 °C), and then mounted on glass slides. Panoramic imaging was performed using a KS-X1500S 3D digital depth-of-field microscope (Nanjing Kaishimai Technology Co., Ltd., Nanjing, China).

### 2.3. Image Processing

SEM images were processed using Microsoft Visio 2024 (Microsoft Corporation, Redmond, WA, USA) to optimize global contrast and unify the background without altering the original morphological features. Antennal parameters were measured using ImageJ software, version 1.54 g (National Institutes of Health, Bethesda, MD, USA).

### 2.4. Statistical Analysis

The data analysis in this study included both quantitative measurements and qualitative observations. Quantitative analysis focused on sensillum length and total antennal length: the total length of six independent antennae from workers, males, and queens was measured, and for each sensillum type, ten individual structures were randomly measured on different antennal segments to obtain dimensional data. Meanwhile, comparisons of sensillum density were based on qualitative observation of SEM images. One-way ANOVA was performed using SPSS 27 software, version 27.0 (IBM Corp., Armonk, NY, USA), and all quantitative data are presented as the mean ± standard error of the mean (mean ± SEM).

### 2.5. Total RNA Extraction and Quality Assessment

Thirty workers, males, and queens of *B. terrestris* were respectively collected. After anesthetizing on ice overlaid with tin foil, their heads (including antennae) were excised, immediately frozen in liquid nitrogen, and stored at −80 °C. Total RNA was extracted from the bumblebee heads using a Trizol reagent kit. Three independent RNA samples were prepared for each caste: each worker sample (designated W_CJ_1 to W_CJ_3) pooled heads from 5 workers, each male sample (D_CJ_1 to D_CJ_3) pooled heads from 5 males, and each queen sample (Q_CJ_1 to Q_CJ_3) pooled heads from 5 queens. RNA purity and concentration were measured using a NanoDrop 2000 spectrophotometer (Thermo Fisher Scientific, Waltham, MA, USA), while RNA integrity was precisely assessed with an Agilent 2100 Bioanalyzer (Santa Clara, CA, USA). Only samples passing quality control were subjected to transcriptome sequencing.

### 2.6. Transcriptome Library Construction and Sequencing

High-quality total RNA that passed quality control was used for mRNA enrichment with oligo(dT)-coupled magnetic beads. The enriched mRNA was fragmented, and first-strand cDNA was synthesized using random primers. Second-strand cDNA synthesis was then performed in a reaction system containing buffer, dNTPs, RNase H, and DNA polymerase I. The resulting double-stranded cDNA products were purified using AMPure XP beads (Beckman Coulter, Inc., Brea, CA, USA). The cDNA ends were blunted using the synergistic action of T4 DNA polymerase and Klenow DNA polymerase, followed by 3′ adenylation and adapter ligation. The ligated products were subsequently size-selected with AMPure XP beads. The second cDNA strand was selectively degraded using Uracil-Specific Excision Reagent (USER) enzyme, and the resulting library was amplified via PCR to construct standardized sequencing libraries. After passing quality inspection, the libraries were sequenced on an Illumina NovaSeq^TM^ 6000 platform (San Diego, CA, USA) using a paired-end 150 bp (PE150) read length configuration for data acquisition. The transcriptome sequencing service was provided by Beijing Novogene Co., Ltd. (Beijing, China), and the raw sequencing data have been deposited in the NCBI SRA database under accession number PRJNA1345014.

### 2.7. Identification of Olfactory Receptor Genes

OR genes were identified by screening functionally annotated and gene description files derived from transcriptomic data for differentially expressed genes annotated as olfactory receptors. Their protein sequences were extracted using TBtools II v2.310 (South China Agricultural University, Guangzhou, China) and subjected to BLASTP (https://blast.ncbi.nlm.nih.gov/Blast.cgi?PROGRAM=blastp&PAGE_TYPE=BlastSearch&LINK_LOC=blasthome, accessed on 26 September 2025) analysis on NCBI to verify full-length coverage and correct original annotation biases. Candidate olfactory receptor gene sequences were subsequently analyzed using the online NCBI ORF Finder (https://www.ncbi.nlm.nih.gov/orffinder/, accessed on 26 September 2025) to identify open reading frames (ORFs). Transmembrane domains were predicted via the TMHMM 2.0 web server (https://services.healthtech.dtu.dk/services/TMHMM-2.0/, accessed on 26 September 2025).

### 2.8. Sequence and Phylogenetic Analysis of Olfactory Receptor Genes

To investigate the evolutionary relationships among the screened differentially expressed OR genes in *B. terrestris*, the previously annotated OR genes from the bumblebee transcriptome, and the ORs of other insects, this study retrieved amino acid sequences of known ORs from 12 Hymenoptera species (including *B*. *terrestris*, *B*. *affinis*, *B*. *huntii*, *B*. *impatiens*, *B*. *pyrosoma*, *B*. *vosnesenskii*, *B*. *bifarius*, *B*. *pascuorum*, *F*. *varia*, *A*. *dorsata*, *A*. *florea*, and *A*. *mellifera*) from the NCBI database. These downloaded amino acid sequences, along with the *B*. *terrestris* OR sequences, were comprehensively aligned using MEGA-7.0.26 software (Temple University, Philadelphia, PA, USA). A phylogenetic tree was subsequently constructed with the neighbor-joining (NJ) method in MEGA 7.0. The visualization and refinement of the phylogenetic tree were performed using the iTOL v7 platform (https://itol.embl.de/, accessed on 26 September 2025).

### 2.9. Quantitative Real-Time PCR Analysis

Quantitative real-time PCR (qPCR) was performed using the same total RNA samples returned by the sequencing company. cDNA was synthesized by reverse transcription using the PrimeScript^TM^ RT reagent kit with gDNA Eraser (Perfect Real Time) (Takara Bio Inc., Shiga, Japan, Cat. No. RR047A). This cDNA served as the template for amplification with the TB Green Premix Ex Taq^TM^ II (Tli RNaseH Plus) (Takara Bio Inc., Shiga, Japan, Cat. No. RR820A). The 10 μL reaction mixture contained: 5 μL of TB Green Premix Ex Taq (Tli RNaseH Plus), 0.5 μL each of forward and reverse primers (10 μmol/L), 500 ng of cDNA, and ddH_2_O to a final volume of 10 μL. The thermal cycling protocol was as follows: 95 °C for 3 min; 40 cycles of 95 °C for 5 s, 57 °C for 30 s, and 72 °C for 30 s. The melting curve was generated under the following conditions: 95 °C for 10 s, followed by 52 °C for 5 s. The bumblebee *s18 Ribosomal* gene (XM_003400778.3) was selected as the internal reference gene for this study [13]. Each sample was analyzed with three biological replicates and three technical replicates. The primer sequences used are listed in Table 1.

## 3. Result

### 3.1. General Antennal Morphology of B. terrestris

**Figure 1:**
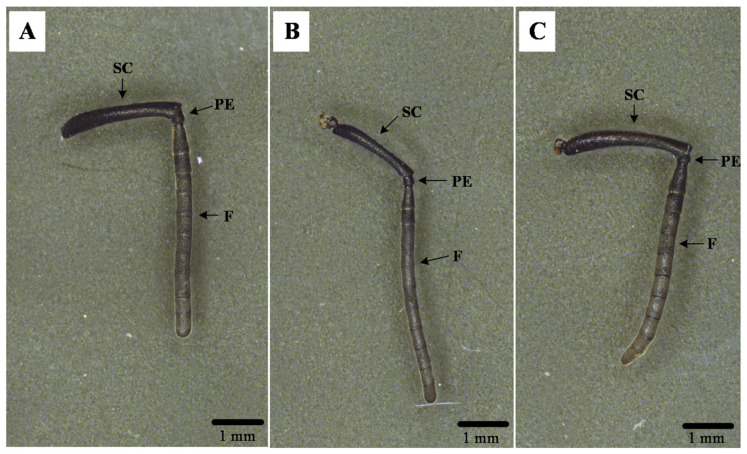
Three-dimensional optical microscopy of *B. terrestris* antennae. Note: (**A**) Worker antenna; (**B**) Drone antenna; (**C**) Queen antenna; SC: Scape; PE: Pedicel; F: Flagellum.

**Figure 2:**
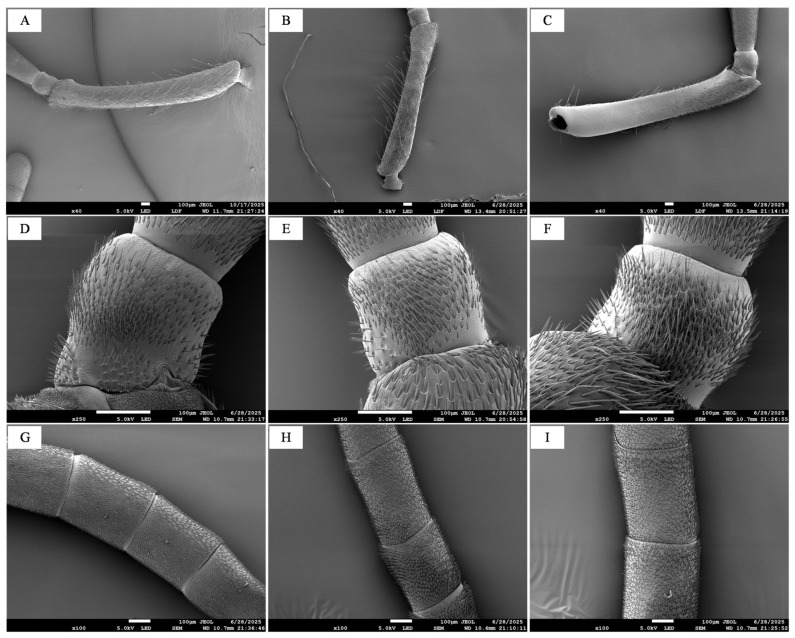
SEM of *B. terrestris* antennae. Note: (**A**) Worker scape; (**B**) Drone scape; (**C**) Queen scape; (**D**) Worker pedicel; (**E**) Drone pedicel; (**F**) Queen pedicel; (**G**) Worker flagellum; (**H**) Drone flagellum; (**I**) Queen flagellum.

The antennae of *B. terrestris* workers, males, and queens are all geniculate in shape, consisting of the scape (SC), pedicel (PE), and flagellum (F). The flagellum comprises 10 flagellomeres in workers and queens, but 11 flagellomeres in males (Figure 1A–C). The total antennal length was 6.61 ± 0.12 mm for workers, 6.20 ± 0.06 mm for males, and 6.72 ± 0.05 mm for queens. No significant difference in length was observed between worker and queen antennae (*p* > 0.05), but both were significantly longer than those of males (*p* < 0.05). The morphology of the scape, pedicel, and flagellum was similar across all three castes, showing no notable differences (Figure 2).

### 3.2. Types of Sensilla on the Antenna of B. terrestri

Observation via SEM revealed the presence of various sensilla on the antenna of *B. terrestris*, including chaetic sensilla (Sch), trichoid sensilla (Str), basiconic sensilla (Sba), placoid sensilla (Spl), coeloconic sensilla (Sco), and Böhm’s bristles (BB).

#### 3.2.1. Sensilla on the Scape

**Figure 3:**
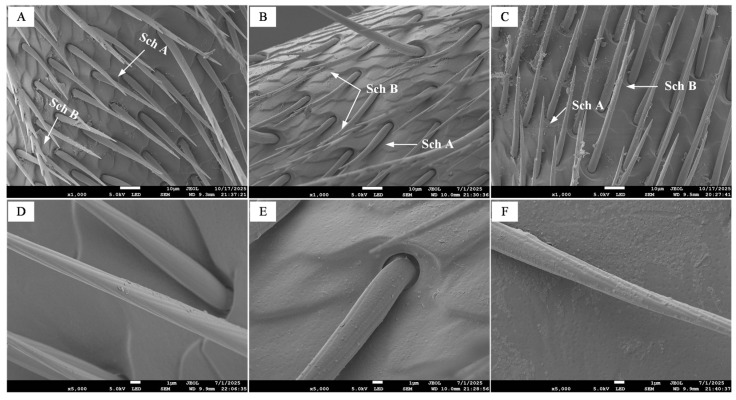
Scanning electron micrographs of scape sensilla in the three castes of *B. terrestris.* Note: (**A**,**D**) Chaetic sensilla of workers; (**B**,**E**) Chaetic sensilla of drones; (**C**,**F**) Chaetic sensilla of queens. Sch A denotes type A chaetic sensilla, while Sch B indicates type B chaetic sensilla.

On the scape of all three castes of *B. terrestris*, only chaetic sensilla were identified, which were categorized into non-branched Sch A and branched, feather-like Sch B. Previous studies have indicated that this type of sensilla possesses dual functionality in both mechanoreception and contact chemoreception [14]. In workers, these sensilla are erect and straight, with Sch A measuring 46.00 ± 2.20 µm (Figure 3A) and Sch B sub-divided by length into Sch B1 (121.04 ± 9.25 µm) and Sch B2 (44.76 ± 1.35 µm). In drones, the sensilla generally curve outward, closely appressed to the antennal wall; Sch A measures 50.09 ± 2.18 µm, while Sch B is classified into Sch B1 (133.44 ± 6.75 µm; Figure 3B) and Sch B2 (61.52 ± 4.91 µm). In queens, the sensilla are partially curved, with Sch A measuring 46.82 ± 1.12 µm and Sch B 72.21 ± 3.87 µm (Figure 3C). No significant difference was observed in Sch A length across castes. Furthermore, the distribution density of Sch A on the scape was similar between workers and queens, both being noticeably higher than that in drones. Sch B was divided into two length-based subtypes in workers and drones but not in queens. However, due to the overall low abundance of Sch B across all three castes, reliable comparison of its density was challenging based on qualitative observation of the available images. All chaetic sensilla exhibited oblique stripes on their surface (Figure 3D–F).

#### 3.2.2. Sensilla on the Pedicel

**Figure 4:**
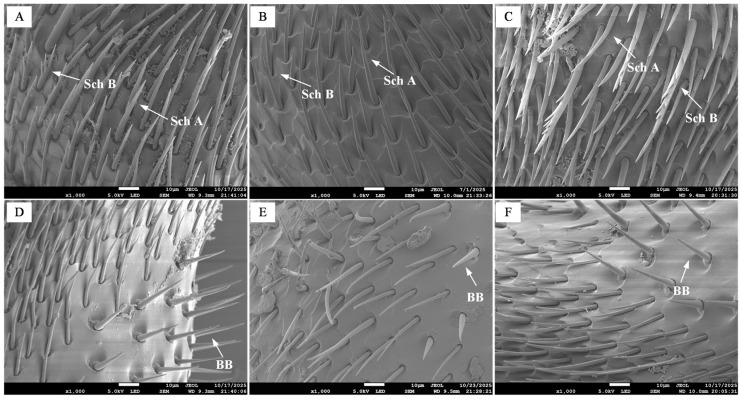
Scanning electron micrographs of chaetic sensilla on the pedicel of *B. terrestris* castes. Note: (**A**,**D**) Worker chaetic sensilla; (**B**,**E**) Drone chaetic sensilla; (**C**,**F**) Queen chaetic sensilla. Sch A denotes type A chaetic sensilla; Sch B indicates type B chaetic sensilla; BB indicates Böhm’s bristles.

**Figure 5:**
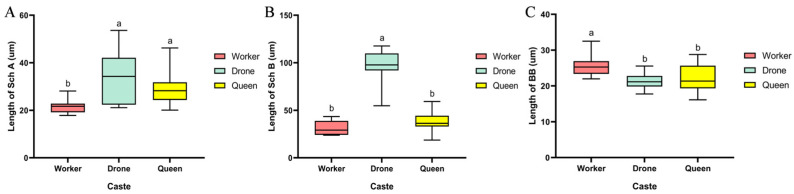
Types and lengths or diameters of sensilla on the antennal pedicel of *B. terrestris*. Note: (**A**) Sch A denotes type A chaetic sensilla; (**B**) Sch B denotes type B sensilla trichodea; (**C**) BB denotes Böhm’s bristles; The data in the table are presented as the mean ± SEM. One-way ANOVA was used to determine significant differences among different castes of *B. terrestris*. Distinct alphabetical notations indicate statistically significant differences among groups (*p* < 0.05). According to Duncan’s multiple range test, means were arranged in descending order for comparison. The highest mean was labeled “a” and compared with other means; those showing no significant difference were also marked “a”. When a significant difference was detected, the subsequent mean was labeled “b”.

On the pedicel of all three castes of *B. terrestris*, two types of chaetic sensilla (Sch A and Sch B) were identified, along with a relatively small number of Böhm’s bristles (Figure 4). Studies have shown that BB can sense antennal position and act as proprioceptors to control its movement [15]. The quantity of Sch B was significantly lower on the pedicel compared to the scape. The measured lengths were as follows: in workers, Sch A measured 21.59 ± 0.93 µm, Sch B was 31.44 ± 2.44 µm, and BB were 25.56 ± 0.96 µm (Figure 4A,D); in drones, Sch A was 33.72 ± 3.33 µm, Sch B was 97.43 ± 5.59 µm, and BB were 21.39 ± 0.74 µm (Figure 4B,E); in queens, Sch A measured 29.16 ± 3.86 µm, Sch B was 38.28 ± 3.61 µm, and BB were 21.95 ± 1.22 µm (Figure 4C,F). Statistical analysis showed no significant difference in Sch A length between drones and queens, but both were significantly longer than in workers (*p* < 0.05) (Figure 5A). The length of Sch B in drones was significantly greater than that in both workers and queens (*p* < 0.05) (Figure 5B). Conversely, the BB in workers were significantly longer than those in both drones and queens (*p* < 0.05) (Figure 5C).

#### 3.2.3. Sensilla on the Flagellum

**Figure 6:**
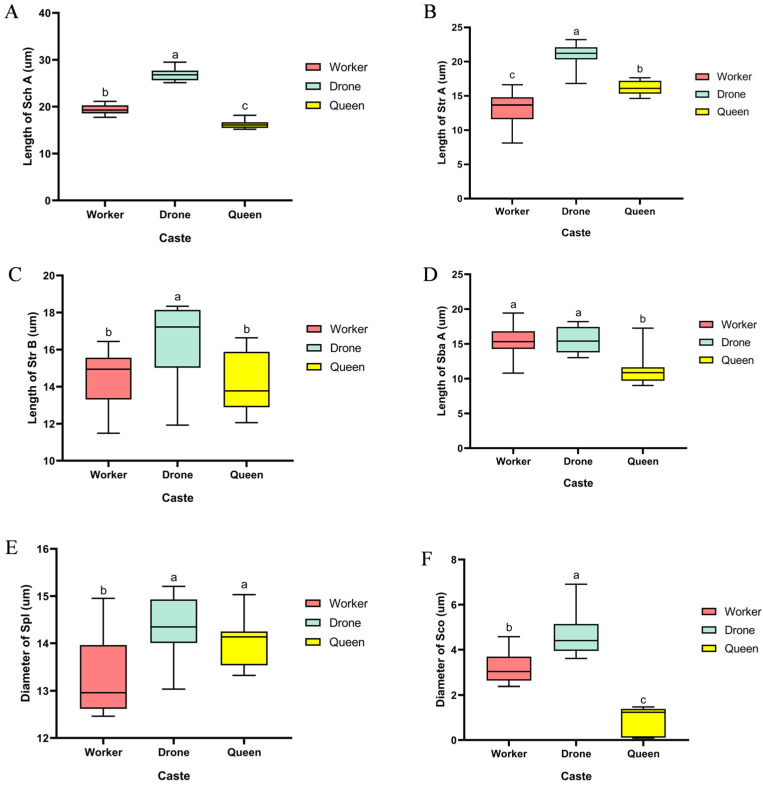
Types and lengths or diameters of sensilla on the antennal flagellum of *B. terrestris*. Note: (**A**) Sch A denotes type A chaetic sensilla; (**B**) Str A denotes type A sensilla trichodea; (**C**) Str B denotes type B sensilla trichodea; (**D**) Sba A denotes type A sensilla basiconica; (**E**) Spl denotes sensilla placodea; (**F**) Sco denotes sensilla coeloconica. The data in the table are presented as the mean ± SEM. One-way ANOVA was used to determine significant differences among different castes of *B. terrestris*. Distinct alphabetical notations indicate statistically significant differences among groups (*p* < 0.05). According to Duncan’s multiple range test, means were arranged in descending order for comparison. The highest mean was labeled “a” and compared with other means; those showing no significant difference were also marked “a”. When a significant difference was detected, the subsequent mean was labeled “b”.

**Figure 7:**
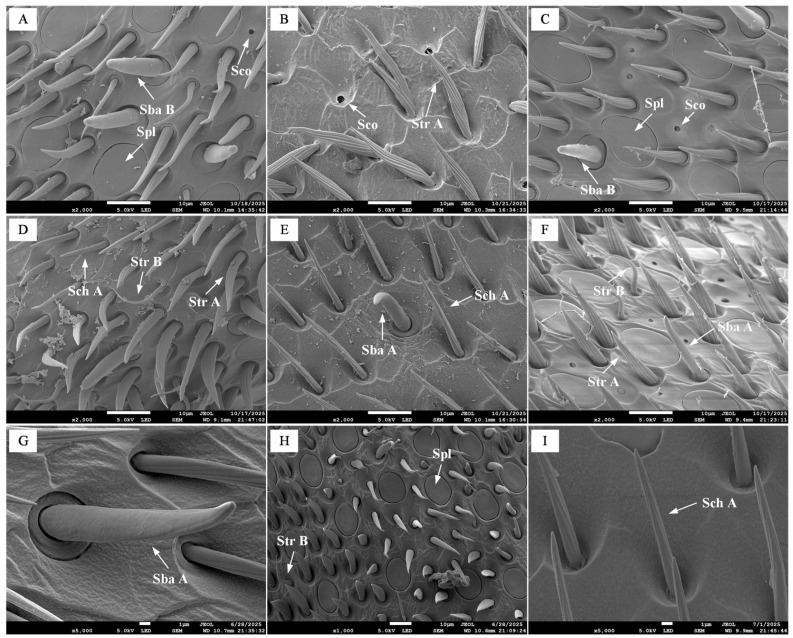
Scanning electron micrographs of sensilla on the flagellum of *B. terrestris* castes. Note: (**A**,**D**,**G**) Sensilla of workers; (**B**,**E**,**H**) Sensilla of drones; (**C**,**F**,**I**) Sensilla of queens. Sch A denotes type A chaetic sensilla; Sch B indicates type B chaetic sensilla; Str A denotes type A sensilla trichodea; Str B denotes type B sensilla trichodea; Sba A denotes type A sensilla basiconica; Sba B denotes type B sensilla basiconica; Sco denotes sensilla coeloconica; Spl denotes sensilla placodea.

On the flagellum of *B. terrestris*, we identified chaetic, trichoid, basiconic, placoid, and coeloconic sensilla, and the lengths or diameters of these sensilla are shown in Figure 6. All three castes possess Sch A, but no feather-like chaetic sensilla were observed (as shown in Figure 7D,E,I). The length of Sch A in drones was significantly greater than that in workers (*p* < 0.05), and worker Sch A was significantly longer than that in queens (*p* < 0.05). All castes have two types of trichoid sensilla, Str A and Str B. Studies have shown that Str serve multiple sensory functions, including mechanoreception and the detection of physical stimuli such as airflow or vibration [16]. Str A exhibits distinct oblique stripes, is slightly wider in the middle, tapers at both ends, and has a pointed tip. Str B bends approximately 90° at its mid-section. The length of Str A in drones was significantly greater than in queens (*p* < 0.05), and queen Str A was significantly longer than worker Str A (*p* < 0.05). Drone Str B was significantly longer than that of both workers and queens (*p* < 0.05). All castes possess Sba A, but Sba B was not found on the drone flagellum. Research has proposed that Sba possess olfactory function [17], while other evidence indicates their potential involvement in microhabitat recognition [18]. Sba B appears generally rounded with a blunt apex, whereas Sba A is relatively flattened. The Sba A in both drones and workers was significantly longer than that in queens (*p* < 0.05), and the Sba B in workers was longer than that in queens, with measured lengths of 12.18 ± 0.86 μm for worker Sba B and 10.40 ± 0.33 μm for queen Sba B. Spl displays a disc-shaped morphology, with a thin groove separating the disc margin from the surrounding cuticle. Previous studies have confirmed that Spl is highly sensitive to queen pheromone components [19]. No significant difference in Spl diameter was observed between drones and queens, but both castes showed significantly larger diameters than workers (*p* < 0.05). The Sco in workers and drones appears as a smooth, hollow pore, while in drones, the Sco is slightly raised around the edges with a hollow center. Studies have shown that Sco play a role in perceiving distant olfactory stimuli [20]. The length of Sco in drones was significantly greater than in workers (*p* < 0.05), and worker Sco was significantly longer than that in queens (*p* < 0.05). Sco Qualitative observations indicate that queens exhibit the highest distribution density of Sco. The diameter of Sco in drones was significantly greater than that in workers (*p* < 0.05), which in turn was significantly larger than that in queens (*p* < 0.05).

### 3.3. Transcriptome Sequencing and Data Analysis

#### 3.3.1. Antennal Transcriptome Sequencing of *B. terrestris*

To determine the gene expression profiles in the heads (including antennae) of the three castes of *B. terrestris*, nine cDNA libraries were constructed, designated as W_CJ_1, W_CJ_2, and W_CJ_3 for workers; D_CJ_1, D_CJ_2, and D_CJ_3 for drones; and Q_CJ_1, Q_CJ_2, and Q_CJ_3 for queens. After filtering, a total of 62.13 Gb of clean reads were obtained, with each sample yielding no less than 5.94 Gb. The G+C content ranged from 38.53% to 41.55%, and the Q30 base percentage was no less than 97.39% in all samples, indicating high data quality suitable for subsequent bioinformatic analysis (Table 2).

#### 3.3.2. Alignment of Transcriptomic Sequences from *B. terrestris* Caste Heads (Including Antennae) to the Bumblebee Reference Genome

The alignment rates of clean reads from all samples to the reference genome ranged between 98.18% and 98.65% (Table 3). Among these, the uniquely mapped reads accounted for 95.59% to 96.29%, with all values exceeding the 70% contamination threshold. The proportion of reads mapped to exonic regions was consistently above 90.21%, while the distribution across genomic features showed: exon > intron > intergenic regions. The proportion of reads in intergenic regions ranged from 2.21% (W_CJ_1) to 4.28% (D_CJ_2), with minimal variation observed between sample types, collectively indicating a high level of completeness in genome annotation.

#### 3.3.3. Enrichment Analysis of Differentially Expressed Genes in Head Transcriptomes Across *B. terrestris* Castes

**Figure 8:**
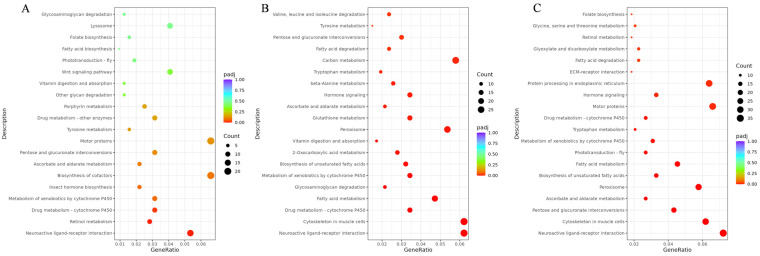
KEGG enrichment results of differentially expressed genes among head transcriptomes of *B. terrestris* castes. Note: (**A**) KEGG enrichment results for worker vs. queen comparison; (**B**) KEGG enrichment results for drone vs. queen comparison; (**C**) KEGG enrichment results for worker vs. drone comparison.

The GO enrichment results of differentially expressed genes (DEGs) from the three pairwise comparisons (worker vs. queen, drone vs. queen, worker vs. drone) revealed distinct functional profiles. In the worker-queen comparison, DEGs were enriched in the cellular component (extracellular region) and molecular function (heme binding) categories (Table S1). The drone-queen comparison showed enrichment in biological process (multicellular organismal process) and molecular function (transmembrane signaling receptor activity) (Table S2). Meanwhile, the worker-drone comparison was enriched in biological process (chitin metabolic process) and molecular function (transporter activity) (Table S3). KEGG pathway analysis consistently identified “Neuroactive ligand-receptor interaction” as the most significantly enriched pathway across all comparisons. Additionally, the worker-queen pair exhibited specific enrichment in retinol metabolism and drug metabolism pathways. The drone-queen pair was uniquely enriched in pathways related to the muscular system (e.g., regulation of actin cytoskeleton) and fatty acid metabolism. The worker-drone pair specifically highlighted pathways involved in pentose conversion, ascorbate metabolism, and peroxisome function (Figure 8A–C).

#### 3.3.4. Screening Results of Differentially Expressed Olfactory Receptor Genes

**Figure 9:**
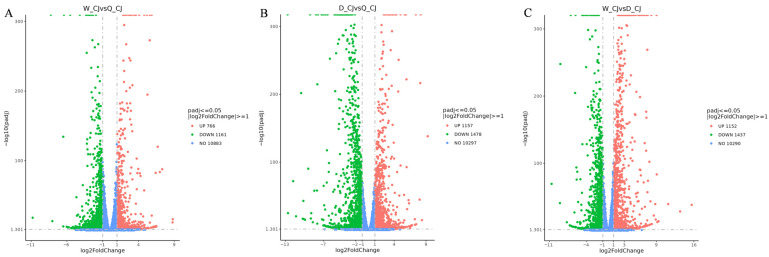
Volcano plots of differentially expressed genes between different comparison groups. Note: (**A**) Volcano plot of differentially expressed genes between workers and queens; (**B**) Volcano plot of differentially expressed genes between drones and queens; (**C**) Volcano plot of differentially expressed genes between workers and drones.

Comparative transcriptomic analysis of *B. terrestris* heads identified a total of 7151 significant differentially expressed genes (DEGs). The detailed distribution included: 1927 DEGs (766 upregulated; 1161 downregulated) in worker-queen comparisons; 2635 DEGs (1157 upregulated; 1478 downregulated) in drone-queen comparisons; and 2589 DEGs (1152 upregulated; 1437 downregulated) in worker-drone comparisons (Figure 9). Through manual screening based on insect olfactory gene annotations, we subsequently identified 16, 35, and 15 olfactory receptor genes from these three DEG sets respectively.

Further analysis focused on eight co-detected OR genes (Table 4): three were identified in the worker vs. queen comparison, all upregulated; seven were found in the drone vs. queen comparison, all upregulated; and three were observed in the worker vs. drone comparison, all downregulated. Sequence analysis revealed that all eight ORs contained seven transmembrane domains and possessed complete open reading frames (ORFs), with lengths ranging from 1125 to 1440 bp.

#### 3.3.5. Phylogenetic Tree of Olfactory Receptor Genes

**Figure 10:**
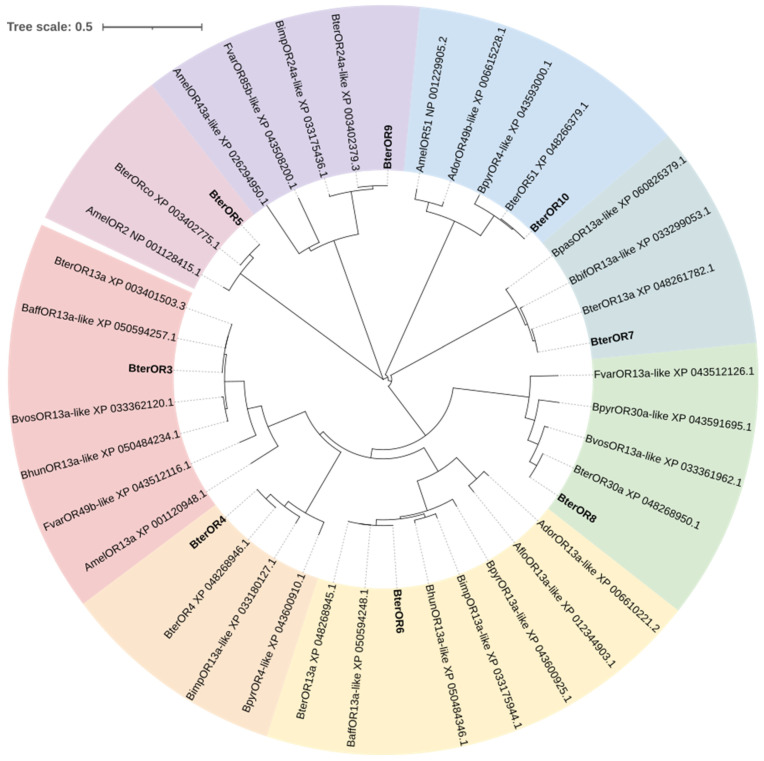
Phylogenetic tree of *B. terrestris* ORs and other hymenopteran ORs constructed using the neighbor-joining method based on amino acid sequences.

The phylogenetic tree constructed by the neighbor-joining method based on amino acid sequences is shown in Figure 10. BterOR5 clustered with the BterORCO co-receptor with high support (Bootstrap = 99%) and formed a co-receptor clade with AmelOR2 of the western honey bee (*Apis mellifera*) with 100% bootstrap support. BterOR4, BterOR8, and BterOR9 each formed fully supported (100%) branches with the annotated *B*. *terrestris* olfactory receptor genes BterOR4, BterOR30a, and BterOR24a-like, respectively. BterOR7 and BterOR13 clustered together (Bootstrap = 99%). Although BterOR13a and BaffOR13a-like showed low internal support (34%), they formed independent branches with BterOR3 and BterOR6, supported by 99% and 100% bootstrap values, respectively. BterOR10 first clustered with BterOR51 (67%) and then merged with BpyrOR4-like with 100% support.

#### 3.3.6. Verification of Olfactory Receptor Gene Expression in *B. terrestris* Heads (Including Antennae) by qRT-PCR

To validate the reliability of the RNA-seq data, we examined eight differentially expressed OR genes using qRT-PCR. Their expression trends were completely consistent with the sequencing data, confirming the reliability of the transcriptome (Table 5).

## 4. Discussions

This study integrated the micromorphological characteristics of antennae from three castes of *B. terrestris* with the expression profiles of OR genes from head transcriptomes. By bridging sensory structures and molecular mechanisms, we systematically revealed the multi-level adaptive evolution of their olfactory system in relation to social division and reproductive strategies.

Our study found that the total antennal length of both workers and queens was significantly greater than that of drones, with queens possessing the longest antennae. Previous research has shown that under identical conditions, antennal length is proportional to body size, suggesting that the longer antennae of queens may be partially attributed to their larger body size [21]. However, this morphological difference is more likely closely associated with their behavioral complexity. Workers are required to perform multiple tasks, including in-nest activities such as brood care and nest maintenance, as well as out-of-nest duties like foraging and collection. Queens, on the other hand, need to execute reproductive behaviors including mating and oviposition. These complex behaviors are highly dependent on a sophisticated sensory system to accurately perceive both internal and external environmental cues. Longer antennae may imply a larger surface area for sensilla distribution and more acute chemosensory capabilities, thereby enhancing the accuracy of behavioral decision-making. In contrast, the primary role of drones is limited to mating with virgin queens, resulting in relatively simpler sensory requirements. This likely has driven the simplification of their antennal structure, reflecting an evolutionary strategy characterized by a “function-structure” trade-off.

Sch were identified on the scape, pedicel, and flagellum of workers, drones, and queens. Previous studies indicate that this type of sensilla possesses dual functionality in both mechanoreception and contact chemoreception [14]. Our study revealed that the length of Sch on all antennal segments of drones was significantly greater than that of workers and queens. This morphological difference likely reflects their heightened need for perceiving chemical or mechanical signals during antennal contact, potentially functioning in mating behavior or the recognition of queen cuticular cues. Compared to the scape, BB were present on the pedicel of all three castes, with workers exhibiting significantly greater bristle length than drones and queens. Previous research has established that BB function as proprioceptors, sensing antennal spatial position and regulating its movement [15]. Consequently, the elongated bristles in workers may enhance their precision in perceiving antennal posture, supporting the more complex flight control demands required for navigating within the nest and hovering between flowers. This indirectly reflects their superior flight capability compared to drones and queens. Str were the most numerous sensilla type on the antennae of *B. terrestris*, distributed exclusively on the flagellum and comprising two subtypes, Str A and Str B. This study found that both subtypes were significantly longer in drones than in workers and queens (*p* < 0.05). These sensilla have been demonstrated to perceive semiochemicals such as sex pheromones and terpenoids [19]. Therefore, the marked elongation of Str in drones increases the sensory surface area, likely enhancing the capture efficiency of queen-emitted sex pheromones. This provides a crucial sensory structural foundation for the precise localization of mates during nuptial flights. Sba sensilla, which are sensitive to general odorants such as plant compounds and pheromones [22], are distributed exclusively on the flagellar segments of the antennae and differ significantly among castes. The length of Sba A was significantly greater in drones than in workers and queens (*p* < 0.05), and no Sba B sensilla were detected in drones. This structural pattern suggests a potential functional specialization in the drone olfactory system: the elongated Sba A sensilla may enhance the detection of long-distance, low-concentration odorant molecules (e.g., queen sex pheromones), while the absence of Sba B could streamline the perception pathways for non-reproduction-related odors, thereby sharpening the olfactory focus on key chemical signals associated with reproduction. In contrast, the presence of Sba B sensilla in workers and queens likely facilitates the perception of a diverse array of odors, such as floral scents, nest odors, or social pheromones, accommodating their broader range of behavioral requirements. Spl have been confirmed to be highly sensitive to queen pheromone components, with drones exhibiting significantly higher behavioral sensitivity to these substances than workers in behavioral experiments [19]. This study found that the diameter of Spl showed no significant difference between drones and queens, but was significantly larger in both castes compared to workers. This structural advantage provides a morphological basis for their high sensitivity to queen pheromones, further supporting the structure-function co-evolution of their olfactory system in fulfilling reproductive tasks. In summary, the caste-specific differentiation in the morphology and quantity of antennal sensilla not only reveals, at the microstructural level, the divergence in olfactory strategies corresponding to “mating specialization” versus “multitask adaptation” in *B. terrestris*, but also provides tangible morphological evidence for the co-evolution of sensory systems and reproductive division of labor in social insects. Across the three castes, the Sco shows complementary adaptive traits: drones display the largest Sco diameter, followed by workers, whereas queens exhibit the smallest diameter but the highest spatial density. Studies indicate that the grooved Sco wall, lacking the middle and inner cuticular layers, may constitute an entry path for odor molecules [23]; moreover, Sco are thought to mediate the detection of olfactory stimuli from long range [20]. Based on these findings, we speculate that the enlarged Sco diameter in drones may enhance the capture efficiency and effective detection distance of key unitary signals such as queen sex pheromones, which could reflect a specialized sensory adaptation strategy. In contrast, queens and workers may adopt a “small-aperture, high-density” design, where numerous Sco are packed into the limited antennal surface to potentially form a high-throughput olfactory surveillance network. This configuration could allow parallel processing of diverse chemical cues—such as floral scents, nest odors, and social pheromones—thereby potentially fitting their more complex and variable behavioral niches.

High-throughput sequencing (RNA-seq) has become a standard method for analyzing gene expression profiles across species and conditions [24]. This study performed transcriptome sequencing on the heads (including antennae) of *B. terrestris* workers, drones, and queens, systematically characterizing the expression profiles of olfactory receptor genes under different sexes and social roles. qRT-PCR validation of eight significantly differentially expressed OR genes showed complete consistency with the sequencing data, confirming data reliability. Pairwise comparisons among the three groups identified 7151 DEGs, from which eight significantly differentiated OR genes were pinpointed. Their expression patterns reflect three distinct biological differentiations: the differences between workers and queens primarily represent task specialization within the same sex; the differences between drones and queens reflect the combined effects of both sex and social role; while the differences between workers and drones highlight fundamental physiological disparities driven by sex-specific reproductive strategies, despite their shared out-of-nest foraging behavior.

In the worker-queen comparison, the significant upregulation of *BterOR3*, *BterOR5*, and *BterOR7* may suggest that workers could have evolved more refined olfactory regulatory capabilities to potentially adapt to efficient out-of-nest foraging. GO enrichment analysis revealed “extracellular region” as the top term in the cellular component category, directly corresponding to the initial stage of olfactory perception—where odorant-binding proteins (OBPs) transport odor molecules across the sensillum lymph and activate ORs on the neuronal membrane to initiate neural signaling [25]. This indicates the coordinated enhancement of genes associated with the extracellular region, establishing a precondition for efficient chemical perception. The concurrent enrichment of “heme binding” and the “Drug metabolism-cytochrome P450” pathway further demonstrates that while enhancing olfaction, workers also upregulate the P450 detoxification system [26] to cope with plant secondary metabolites in nectar and pollen, as well as potential pesticide residues. This integrated adaptation enables a seamless “perception-intake-detoxification” process.

In the drone-queen comparison, a total of seven OR genes (*BterOR3*, *BterOR4*, *BterOR5*, *BterOR6*, *BterOR7*, *BterOR8*, and *BterOR9*) were upregulated in drones, representing the highest number of upregulated genes among all comparison groups. This pattern may suggest a high degree of specialization in their olfactory system, which could align closely with the drone’s primary biological mission of mating [27]. Unlike queens, which remain in the nest throughout their lives, drones must detect the extremely low concentrations of sex pheromones released by virgin queens in open outdoor environments [28]. Consequently, this large-scale upregulation of olfactory receptor genes could be interpreted as an adaptive evolutionary response at the olfactory level to potentially maximize their reproductive success. The most significantly enriched term in the GO molecular function category, “transmembrane signaling receptor activity,” indicates that the large-scale upregulation of olfactory receptor genes in drones is not an isolated event, but rather a concentrated manifestation of adaptive evolution within the entire transmembrane signaling perception system. The ultimate objective is to achieve efficient and precise recognition of mating signals. Conversely, the low expression of olfactory receptor genes in queens may correspond to their ecological niche as “in-nest egg-laying machines”. Studies have shown that increased larval pheromones within the nest prompt worker bees to extend their feeding time to the queen, thereby enhancing her oviposition rate [29]. Within this social structure, which heavily relies on workers for information transfer, the queen has no need to maintain a complex olfactory receptor system for direct perception of external odors. Therefore, the down-regulation of OR expression likely represents an energy-saving adaptation to the queen’s caste-specific role as an in-hive “egg-laying specialist”: by minimizing the metabolic cost of maintaining an extensive olfactory receptor repertoire, she can reallocate resources to oogenesis and oviposition, while relying on worker-mediated information transfer for within-nest chemical communication, thereby maximizing overall reproductive efficiency. Within the drone upregulation profile, the expression levels of *BterOR3* and *BterOR4* were the most prominent. It is hypothesized that these two receptors possess ultra-high affinity for key components of the queen sex pheromone, serving as core molecules mediating the capture of long-distance, low-concentration signals. This represents a crucial evolutionary strategy for drones to maximize their reproductive success.

Although both workers and drones exhibit “out-of-nest” behavior, the underlying motivations are fundamentally distinct: workers leave the nest to forage for nectar sources [30], whereas drones do so to mate with queens and reproduce [31]. This fundamental difference drives their olfactory systems toward opposite evolutionary poles: “generalization” versus “specialization.” In the worker-drone comparison, three olfactory receptor genes (*BterOR3*, *BterOR4*, and *BterOR10*) were significantly upregulated in drones. Notably, the expression levels of *BterOR3* and *BterOR4* in drones were significantly higher than those in both workers and queens. This suggests that drones may concentrate their perceptual resources on a limited number of highly specific receptors, forming a “minimalist yet specialized” sex pheromone detection module. In contrast, workers must utilize the same olfactory “hardware” to perform diverse tasks such as brood care, nest maintenance, and foraging from various floral sources. Consequently, their OR expression profile must maintain a “broad and plastic” balanced state [32]. Behavioral polymorphism has been confirmed as a major driver of brain gene expression in bees [33]. The multi-task, multi-stage behavioral division of labor in workers necessitates that their olfactory receptor expression profile retains a degree of “balance” and “plasticity” to flexibly respond to varying sensory demands. Therefore, workers maintain only basal expression levels of specific pheromone receptors, while drones adopt an “overexpression–ultra-high affinity” strategy to amplify mating signals. This contrast in molecular-level expression mechanistically explains how the same out-of-nest behavior, driven by divergent survival objectives, can shape fundamentally distinct evolutionary trajectories in the olfactory system.

The phylogenetic tree clusters BterOR5 within the same clade as AmelOR2 from *Apis mellifera* (100% bootstrap support) and BterORco (99% support), suggesting its potential role as a conserved co-receptor in *B. terrestris* (Figure 7). Previous studies have established that AmelOR2, the ortholog of DmelOR83b, functions as an essential auxiliary subunit in functional olfactory receptor complexes and must be co-expressed with the specific receptor AmelOR1 to mediate sensitive responses to the queen pheromone component 9-ODA [34]. Although BterOR5 shows high sequence similarity with AmelOR2 (91.63% identity), and thus may serve an analogous co-receptor role in pheromone detection, it should be noted that their expression patterns in homologous olfactory neurons remain unverified. Based on this phylogenetic conservation, we hypothesize that key pheromone receptors in *B. terrestris* may similarly require assembly with BterOR5 into heteromeric complexes to achieve full functionality, positioning this gene as a candidate target for future “function-ligand” screening studies. BterOR4 showed 100% identity with its reference sequence, confirming the reliability of its annotation. BterOR7, BterOR8, and BterOR9 clustered with BterOR13, BterOR30a, and BterOR24a-like, respectively, with extremely high bootstrap support. However, their amino acid sequence identity was below 85%, suggesting that they originated from recent gene duplication events followed by rapid divergence. This pattern is consistent with the “birth-and-death” evolutionary model observed in insect olfactory receptor (OR) families [35,36]. BterOR3 and BterOR6 formed two distinct, deeply branching clades (with 99% and 100% bootstrap support, respectively) with different accessions of BterOR13a and BaffOR13a-like. This topology reveals that they diverged synchronously following ancestral gene duplication during the radiation of the *Bombus* genus, representing a typical “radiation-retention” evolutionary pattern [37]. BterOR10 and BterOR51 clustered together with moderate support within a highly supported branch containing BpyrOR4-like, suggesting they belong to a conserved OR subfamily within *Bombus*. Their functions may exhibit overlap or redundancy. We therefore hypothesize that the function of BterOR10 is likely associated with other members of this subfamily.

Although this study provides comprehensive evidence for caste-specific olfactory adaptation in *B. terrestris*, several limitations should be noted. First, the association between upregulated BterOR3/5/7 expression and worker foraging behavior is primarily based on cross-caste comparative evidence. While this research design effectively elucidates the influences of sex and social roles, incorporating more multidimensional validation in the future would be more persuasive. Second, due to sampling constraints, this study does not include transcriptomic dynamic data from different developmental stages of worker bees. Subsequent research incorporating temporal expression profiles of newly emerged workers and mature foragers would more clearly reveal the developmental temporal characteristics of OR gene expression regulation. Furthermore, the scanning electron microscopy analysis has certain limitations: the assessment of sensilla density in this study was primarily based on qualitative observation, lacking systematic quantitative statistical analysis. Future research should employ image-based morphometric methods to accurately quantify sensilla density through standardized counting and spatial distribution analysis. Additionally, although BterOR5 and AmelOR2 show high sequence similarity, the conservation of their expression patterns in olfactory neurons still requires direct validation through experiments such as in situ hybridization. These limitations indicate directions for future research, including functional validation through gene knockdown, establishment of gene expression profiles at different behavioral stages, and comparative studies of co-receptor expression patterns across species.

Integrating antennal ultrastructure with high-throughput expression data, this study constructs a complete chain of evidence for “morphology-molecule” co-evolution in *B. terrestris*. Drones adopt a “quality-priority” olfactory strategy—characterized by longer sensilla trichodea and basiconica, larger-diameter sensilla coeloconica, and the high-level expression of seven OR genes—to form a long-range, high-sensitivity detection system specialized for the singular signal of queen sex pheromones. In contrast, workers and queens follow a “quantity-breadth priority” pathway. By retaining a complete repertoire of sensilla types (e.g., Sba B), increasing the density of sensilla coeloconica, and maintaining a relatively balanced OR expression profile, they construct a high-throughput chemical monitoring network capable of simultaneously recognizing floral scents, nest odors, and social pheromones. The high congruence between sensilla morphology and receptor expression across castes reveals, for the first time, how the olfactory system of *B. terrestris* undergoes coordinated differentiation at both structural and genetic levels. This precise adaptation aligns with their distinct ecological roles—specialized mating versus complex social tasks—thereby establishing a new multi-level paradigm for understanding sensory-behavioral co-evolution in social insects.

## 5. Conclusions

This integrated study of antennal ultrastructure and transcriptomic profiling in bumblebees provides evidence for caste-specific evolution of the olfactory system. Our findings suggest two distinct adaptive patterns; drones exhibit characteristics consistent with a specialized “quality-priority” strategy, including elongated chemosensory sensilla and selective upregulation of key olfactory receptors, which are features that may support high-sensitivity detection of queen sex pheromones. In contrast, workers and queens display traits aligned with a generalized “quantity-breadth priority” strategy, maintaining diverse sensilla types, high sensilla density, and a balanced olfactory receptor expression profile that may facilitate broad-spectrum chemical monitoring capability. The correspondence observed between morphological traits and molecular expression patterns across castes suggests the potential existence of a “morphology-molecule” co-evolutionary pathway, which appears to be influenced by their respective reproductive functions and social roles. This study proposes a multi-level research framework for exploring sensory-behavioral coevolution in social insects.

## Figures and Tables

**Table 1:** Primer Information.

Primer Name	Forward Primer Sequence	Reverse Primer Sequence
*S18*	AGCGTGCTGGAGAATGTTCA	TCGTTCCAAGTCCTCACGAAG
*BterOR3*	AGGTCGAGACGAGCCTGTTA	TCCACGGTTCGCTGTTTTA
*BterOR4*	TGTCGCCTTACTACGAAATGAT	CCTGTCGCAGAAAGTGATGAG
*BterOR5*	ACTCTTTTGCTCGTGGCTCC	TCTTTGTCAATCCATTCGGTC
*BterOR6*	CCCTTATTTGTTACGCCCTTAG	AATACCGATTCCAGTTTTCCAC
*BterOR7*	TTGAATGAAGCGACCGAATG	CAAACTTGCCCGAACCGT
*BterOR8*	ATGCCCCGACAACTTCTTATG	ATCGTCCACCATCCCGAGT
*BterOR9*	GAAACGCAACAGTATGAAAAGG	TTCCACCAAAAGTCAAGACAAG
*BterOR10*	AACTCGGTCGTTGCGTCA	GCCTTCCCATCCTTCTTCA

**Table 2:** Statistical evaluation of transcriptome data from the heads (including antennae) of *B. terrestris* castes.

Samples	Number of Clean Reads	Number of Clean Bases	G+C (%)	Q30 (%)
W_CJ_1	48,904,316	7.34 G	41.31	97.6
W_CJ_2	45,553,148	6.83 G	40.85	97.39
W_CJ_3	40,274,634	6.04 G	41.03	97.56
D_CJ_1	49,397,130	7.41 G	39.22	97.53
D_CJ_2	47,151,224	7.07 G	38.53	97.7
D_CJ_3	39,613,776	5.94 G	38.96	97.69
Q_CJ_1	49,503,648	7.43 G	41.55	97.65
Q_CJ_2	44,831,868	6.72 G	40.8	97.58
Q_CJ_3	48,984,808	7.35 G	40.87	97.43

Note: W_CJ_1–3: Three worker head (including antennae) samples of bumblebee, with three replicates; D_CJ_1–3: Three drone head (including antennae) samples of bumblebee, with three replicates; Q_CJ_1–3: Three queen head (including antennae) samples of bumblebee, with three replicates.

**Table 3:** Alignment statistics of transcriptomic sequences from *B. terrestris* caste heads (including antennae) against the reference genome.

Samples	Number of Clean Reads	Percentage of the Clean Reads Mapped to the Reference Genome	Percentage of the Clean Reads Mapped to Unique Site on the Reference Genome	Percentage of Read Segments Aligned to Exonic Regions Compared to the Total Number of Read Segments	Percentage of Read Segments That Align to the Intronic Regions Compared to the Total Number of Read Segments	Percentage of Read Segments Aligned to Intergenic Regions Compared to the Total Number of Read Segments
W_CJ_1	48,904,316	98.65	95.96	94.06	3.73	2.21
W_CJ_2	45,553,148	98.33	95.82	93.75	3.86	2.39
W_CJ_3	40,274,634	98.33	95.72	93.77	3.82	2.41
D_CJ_1	49,397,130	98.37	96.29	91.00	5.28	3.72
D_CJ_2	47,151,224	98.18	95.59	90.21	5.51	4.28
D_CJ_3	39,613,776	98.20	95.98	90.91	5.24	3.84
Q_CJ_1	49,503,648	98.52	96.10	92.74	4.93	2.33
Q_CJ_2	44,831,868	98.39	96.01	92.21	5.13	2.66
Q_CJ_3	48,984,808	98.45	96.12	92.45	5.01	2.54

Note: W_CJ_1–3: Three worker head (including antennae) samples of bumblebee, with three replicates; D_CJ_1–3: Three drone head (including antennae) samples of bumblebee, with three replicates; Q_CJ_1–3: Three queen head (including antennae) samples of bumblebee, with three replicates.

**Table 4:** Expression Profiles of Significantly Differentially Expressed Genes in Different Comparison Groups.

Sig. DEG	W vs. Q	D vs. Q	W vs. D
*BterOR3*	up	up	down
*BterOR4*		up	down
*BterOR5*	up	up	
*BterOR6*		up	
*BterOR7*	up	up	
*BterOR8*		up	
*BterOR9*		up	
*BterOR10*			down

Note: Sig. DEG denotes significantly differentially expressed genes; W vs. Q represents the worker vs. queen comparison group of bumblebees; D vs. Q represents the drone vs. queen comparison group; W vs. D represents the worker vs. drone comparison group; Up indicates upregulated genes; Down indicates downregulated genes.

**Table 5:** qRT-PCR validation of differentially expressed genes among *Bombus terrestris* castes.

Gene Name		Log2 Fold Change by RNA-Seq	Log2 Fold Change by qRT-PCR
BterOR3	W vs. Q	1.85	0.74
	D vs. Q	3.09	5.11
	W vs. D	−1.27	−4.38
BterOR4	D vs. Q	4.26	7.66
	W vs. D	−1.96	−3.15
BterOR5	W vs. Q	8.80	10.27
	D vs. Q	8.36	10.30
BterOR6	D vs. Q	4.75	14.09
BterOR7	W vs. Q	3.33	14.23
	D vs. Q	3.08	14.54
BterOR8	D vs. Q	3.59	14.11
BterOR9	D vs. Q	4.54	14.65
BterOR10	W vs. D	−1.36	−4.77

Note: W vs. Q represents the worker vs. queen comparison group in *Bombus terrestris*; D vs. Q represents the drone vs. queen comparison group; W vs. D represents the worker vs. drone comparison group. All samples used for qRT-PCR were derived from those subjected to transcriptome sequencing. Each biological replicate consisted of 10 *B. terrestris* heads (including antennae), and the analysis included three biological replicates and three technical replicates for each sample.

## Data Availability

The data presented in this study are openly available in [NCBI] at [https://www.ncbi.nlm.nih.gov/bioproject/PRJNA1345014], reference number [PRJNA1345014].

## Supplementary Materials

The following supporting information can be downloaded at: https://www.mdpi.com/article/10.3390/insects17010055/s1, Table S1: GO Enrichment Analysis of Differentially Expressed Genes in Heads (Including Antennae) Between Workers and Queens; Table S2: GO Enrichment Analysis of Differentially Expressed Genes in Heads (Including Antennae) Between Drones and Queens; Table S3: GO Enrichment Analysis of Differentially Expressed Genes in Heads (Including Antennae) Between Workers and Drones.
