# Supplementary material for: Integrative Analysis of Antennal Morphology and Olfactory Receptor Gene Expression Across the Three Castes of Bombus terrestris (Hymenoptera: Apidae)
Source: Insects. 2026 Jan 1;17(1):55. doi: 10.3390/insects17010055 (PMC12841855; doi:10.3390/insects17010055)
Supplement: Supplementary file 1 [file insects-17-00055-s001.zip › insects-4001694-supplementary.pdf]

Table S1. GO Enrichment Analysis of Differentially Expressed Genes in the Head (Including Antennae) Between Worker and Queen of *Bombus terrestris*.

GO category	Term type	GO code	Number	P-value
extracellular region	CC	GO:0005576	19	9.05E-05
heme binding	MF	GO:0020037	26	8.63E-09
tetrapyrrole binding	MF	GO:0046906	26	1.30E-08
iron ion binding	MF	GO:0005506	22	4.90E-07
oxidoreductase activity, acting on paired donors, with incorporation or reduction of molecular oxygen	MF	GO:0016705	22	4.90E-07
transmembrane signaling receptor activity	MF	GO:0004888	56	9.23E-07
signaling receptor activity	MF	GO:0038023	56	1.24E-06
molecular transducer activity	MF	GO:0060089	56	1.24E-06
signal transducer activity	MF	GO:0004871	56	1.44E-06
odorant binding	MF	GO:0005549	37	6.68E-06
cofactor binding	MF	GO:0048037	46	2.65E-05
olfactory receptor activity	MF	GO:0004984	31	0.000160209
oxidoreductase activity	MF	GO:0016491	55	0.000213288
G-protein-coupled receptor activity	MF	GO:0004930	21	0.001612806
chitin binding	MF	GO:0008061	13	0.002050831

Table S2. GO Enrichment Analysis of Differentially Expressed Genes in the Head (Including Antennae) Between Drone and Queen of *Bombus terrestris*.

GO category	Term type	GO code	Number	P-value
multicellular organismal process	BP	GO:0032501	56	5.28E-07
sensory perception of smell	BP	GO:0007608	47	2.33E-06
system process	BP	GO:0003008	49	6.73E-06
sensory perception	BP	GO:0007600	49	6.73E-06
sensory perception of chemical stimulus	BP	GO:0007606	49	6.73E-06
nervous system process	BP	GO:0050877	49	6.73E-06
G-protein-coupled	BP	GO:0007186	36	2.35E-05

receptor signaling pathway				
transmembrane transport	BP	GO:0055085	74	0.000194712
extracellular region	CC	GO:0005576	23	0.000919553
transmembrane signaling receptor activity	MF	GO:0004888	93	5.49E-15
signaling receptor activity	MF	GO:0038023	93	1.02E-14
molecular transducer activity	MF	GO:0060089	93	1.02E-14
odorant binding	MF	GO:0005549	58	2.64E-10
G-protein-coupled receptor activity	MF	GO:0004930	37	3.85E-08
iron ion binding	MF	GO:0005506	28	6.52E-08

Table S3. GO Enrichment Analysis of Differentially Expressed Genes in Heads (Including Antennae) Between Workers and Drones of *Bombus terrestris.*

GO category	Term type	GO code	Number	P-value
chitin metabolic process	BP	GO:0006030	23	2.55E-07
amino sugar metabolic process	BP	GO:0006040	23	2.55E-07
glucosamine-containing compound metabolic process	BP	GO:1901071	23	2.55E-07
aminoglycan metabolic process	BP	GO:0006022	24	4.64E-07
G-protein-coupled receptor signaling pathway	BP	GO:0007186	37	7.98E-06
transmembrane transport	BP	GO:0055085	76	4.53E-05
transport	BP	GO:0006810	121	0.000148392
establishment of localization	BP	GO:0051234	121	0.00016406
localization	BP	GO:0051179	122	0.000276451
drug metabolic process	BP	GO:0017144	27	0.000326994
ion transport	BP	GO:0006811	47	0.000518992
cyclic nucleotide metabolic process	BP	GO:0009187	9	0.000561237

cyclic nucleotide biosynthetic process	BP	GO:0009190	9	0.000561237
extracellular region	CC	GO:0005576	26	1.50E-05
transporter activity	MF	GO:0005215	104	1.99E-13
